# NC CLASP: the structure and reach of a statewide antibiotic stewardship education program

**DOI:** 10.1017/ash.2025.10245

**Published:** 2025-12-17

**Authors:** Christine E. Kistler, Elizabeth S. Thomas, Evelyn Cook, Danielle Doughman, Chineme Enyioha, C. Adrian Austin, Mallory McClester Brown, Zachary I. Willis, James W. Johnson, Saif Khairat, Phillip D. Sloane

**Affiliations:** 1 Division of Geriatric Medicine, School of Medicine, University of Pittsburgh, Pittsburgh, PA, USA; 2 North Carolina Statewide Program for Infection Control and Epidemiology, Chapel Hill, NC, USA; 3 Carolina Antimicrobial Stewardship Program, University of North Carolina Medical Center, Chapel Hill, NC, USA; 4 Department of Family Medicine, University of North Carolina, Chapel Hill, NC, USA; 5 Division of Geriatric Medicine, University of North Carolina, Chapel Hill, NC, USA; 6 Division of Pulmonary and Critical Care Medicine, University of North Carolina, Chapel Hill, NC, USA; 7 Department of Pediatric Medicine, University of North Carolina, Chapel Hill, NC, USA; 8 Division of Practice Advancement and Clinical Education, UNC Eshelman School of Pharmacy, University of North Carolina at Chapel Hil, Chapel Hill, NC, USA; 9 School of Nursing, University of North Carolina, Chapel Hill, NC, USA

## Abstract

Experts conducted a free statewide series on antibiotic stewardship for hospital, outpatient, and long-term care settings. In total, 366 participants from 244 sites represented 66% of counties in the state. Furthermore, 62% worked in nonmetropolitan counties, and 55% were from counties with medium-to-high social vulnerability, demonstrating reach into diverse sites.

## Introduction

For two decades, the US Centers for Disease Control and Prevention (CDC) has identified antibiotic stewardship as a key priority in the fight against antimicrobial resistance.^1^ As part of this effort, hospital-based antimicrobial stewardship programs markedly increased from 41% in 2015 to 84% in 2019.^2^ Greater implementation of antimicrobial stewardship in hospitals, combined with improvements in infection prevention, was associated with an 18% decrease in death rate due to antibiotic resistance.^2^ Unfortunately, the COVID-19 pandemic was associated with a profound decline in antibiotic stewardship and a resurgence in widespread antibiotic overuse.^3^

In response to the overuse trends observed from the COVID-19 pandemic and continued concern over antibiotic resistance, the CDC and the American Rescue Plan of 2021 provided state departments of health with funds to refocus efforts on antimicrobial resistance. In 2022, as part of that effort, the North Carolina Department of Health and Human Services, University of North Carolina, and Statewide Program for Infection Control and Epidemiology (SPICE) created the North Carolina Clinical Antibiotic Stewardship Partners (NC CLASP). Its goal was to promote antibiotic stewardship resources for staff working in acute care hospitals, outpatient clinics, and long-term care (LTC) settings across the state of North Carolina. We hypothesized that an intervention similar to the ECHO model would reach a large number of sites.

## Methods

### Participants

NC CLASP recruited participants from across the state in cohorts, with each cohort being offered a series of programs and materials. For hospital and LTC participant recruitment, SPICE used prior lists of participants and existing listservs. For outpatient clinics, a presentation was made to a clinic network affiliated with a large statewide healthcare system. Invited professionals included nurses, infection preventionists, nursing home administrators, pharmacists, and physicians. All participation was voluntary, and staff roles were not collected.

### Intervention

The content was delivered to acute care hospitals, outpatient clinics, and long-term care settings via a series of webinars, statewide in-person conferences, regional in-person “lunch and learn” sessions, and evidence-based resources on the NC CLASP website. NC CLASP was active from December 2022 to July 2024. Each session offered educational credits to nurses, pharmacists, and physicians. The webinars included didactics about the core elements and quality improvement (QI) principles and used small group and large group discussions, polls, and role plays to encourage participant engagement.

### Measures

Participants were asked about basic demographic characteristics, including their professional role and participating site. Additional variables obtained for each site included the social vulnerability index (SVI) and rurality of their sites. The SVI indicates the relative vulnerability of every US county across 15 social factor groups with a composite value ranging from 0 to 1, where 1 is the highest level of social vulnerability.^4^ Rurality was defined as non-metropolitan counties.^5^ In addition, we determined whether participant hospitals were critical access hospitals^6^ and used CMS Nursing Home Compare to determine CMS Star rating for participant nursing homes.^7^

### Analysis

We computed descriptive statistics, including SVI and rurality, for each cohort and the group overall. To assess reach, we used the SVI maps for North Carolina to determine the geographic distribution of all sites. We then conducted a review of session content and reported the frequency of different intervention components across sessions.

## Results

### Participant characteristics

Across North Carolina, 366 participants from 244 healthcare sites joined sessions for at least one of the three clinical settings (Table 1). Most hospitals in both cohorts (93% in total) were non-academic, and 65% (11 of 17 sites) of cohort 1 and 46% (12 of 26 sites) of cohort 2 were in counties with medium-to-high SVI (Supplemental Figure). In cohort 1, 26 of 31 participants attended at least 50% of sessions. Among outpatient clinics, in cohort 1, 5 clinics (42%) were in counties with medium-to-high SVI, and 18 (42%) in cohort 2. In cohort 1, 75% (15/20) participants completed at least 60% of the series. Of 210 LTC participating sites, 113 (54%) were from counties with medium-to-high SVI. In cohort 1, 12 of 22 participants completed at least 60% of the series. Of the 422 LTC communities in North Carolina, 49.8% (210/422) were reached with at least one session.

**Table 1.** NC CLASP participant characteristics by location, clinical setting, and cohort

Participant characteristics	*N* (%) of mean (SD)			
**All clinical settings**				
Total participating sites	244			
Number of staff/employees participating	336			
Counties in state represented by ≥1 program participants	66 (66%)			
Sites located in nonmetropolitan/rural counties	151 (62%)			
Sites in counties with medium- to high-SVI scores (SVI > 0.5)	135 (55%)			
**Hospitals, eligible *N* = 102#x002A;**	**Cohort 1, *N* = 17**		**Cohort 2, *N* = 26**	
Critical access hospitals, eligible *N* = 20#x002A;#x002A;	2 (12%)		3 (15%)	
Hospitals in counties with medium- to high-SVI	11 (65%)		12 (46%)	
Non-academic settings	16 (94%)		24 (92%)	
Staff/employees participating	31		34	
Sessions attended, average (of total)	2.6 (of 4)		1.5 (of 6)	
Staff/employees who completed >/=50% of series	26 (84%)		0	
**Outpatient clinics, *N* #x002A;#x002A;#x002A;**	**Cohort 1, *N* = 12**		**Cohort 2, *N* = 43**	
Clinics in counties with medium- to high-SVI	5 (42%)		18 (42%)	
Staff/employees participating	20		47	
Sessions attended, average (of total)	2.2 (of 3)		1.5 (of 9)	
Staff/employees who completed >60% of series	15 (75%)		0	
**Long-term Care (LTC) Communities, eligible *N* = 422#x002A;#x002A;#x002A;#x002A;**	**Cohort 1, *N* = 14**	**Cohort 2, *N* = 130**		**Cohort 3, *N* = 66**
Average CMS Overall Star Ratings, from 2022–2023#x002A;#x002A;#x002A;#x002A;#x002A;	3.5	2.8		2.7
LTC communities from counties with medium- to high-SVI	7 (50%)	70 (54%)		36 (55%)
Staff/employees participating	22	141		71
Sessions attended, average (of total)	4.5 (of 8)	1 (of 8)		1.7 (of 8)
Staff/employees who completed >60% of series	12 (55%)	0		0

**#x002A;:** As of 1/1/2023, there were 102 hospitals in the state. https://www.shepscenter.unc.edu/programs-projects/rural-health/list-of-hospitals-in-the-u-s/

**#x002A;#x002A;:** North Carolina had 20 critical access hospitals. https://info.ncdhhs.gov/dhsr/ahc/pdf/criticalaccess.pdf

**#x002A;#x002A;#x002A;:** Total number of outpatient clinics in North Carolina is unknown and therefore data cannot be presented as a percentage of the total.

**#x002A;#x002A;#x002A;#x002A;:** There were 422 long-term care facilities, from NC Division of Health Service Regulation Licensed Facilities; Nursing Home Listing Alphabetical, updated 5/8/24 https://info.ncdhhs.gov/dhsr/reports.htm

**#x002A;#x002A;#x002A;#x002A;#x002A;:** Not all nursing homes in cohorts had a star rating found on CMS Compare

### Intervention delivery

Core elements of antibiotic stewardship were presented in the sessions for all cohorts from each setting (Table 2). One cohort from each setting had sessions focused on quality improvement principles. All webinars across all cohorts used small group discussions. No hospital or LTC pursued the option to have individual coaching sessions or QI support. Individual antibiotic stewardship topics varied across cohort and setting. Two statewide in-person conferences were held at the end of cohort 1 and near the end of the last cohort (November 2023 and May 2024) and were open to NC CLASP participants from all settings at no cost. Topics differed between the two conferences, but both included plenary sessions focused on wider antimicrobial stewardship issues and setting-specific topics. Both conferences offered continuing education credits and breakfast and lunch. Three additional “Lunch and Learn” sessions were held in each of the three primary regions in the state (the mountains, piedmont, and coastal area). Participants from all settings were invited to attend. A website was developed to house the educational content from all sessions and included a variety of evidence-based resources and treatment guidelines.

**Table 2.** Intervention delivery across clinical setting and cohort

Intervention Components	Clinical Focus						
	Hospital		Outpatient		Long-term care#x002A;		
	Cohort 1	Cohort 2	Cohort 1	Cohort 2	Cohort 1	Cohort 2	Cohort 3
Core element sessions	4	6	1.5	9	8	8	8
Other elements							
Quality improvement	0	1	1.5	1	7	7	7
Large group discussions	3	2	0	9	6	6	6
Small group discussions	4	5	3	0	7	7	7
Participant demonstrations	0	0	0	0	2	0	0
Role-play	0	0	0	0	1	1	1
Antibiotic stewardship topics	4	6	9	11	2	10	6
In-person meetings							
Regional session “lunch and learn”	3						
Statewide conference	2						
Evidence-based resources	Included Centers for Disease Control and Prevention, Agency for Healthcare Research and Quality, AMDA resources, etc.						
Evidence-based treatment guidelines	Included Centers for Disease Control and Prevention, Agency for Healthcare Research and Quality, AMDA resources, etc.						
Website	https://spice.unc.edu/						

Team characteristics	Clinical focus		
	Hospital, *N* = 2	Outpatient, *N* = 2	Long-term care, *N* = 7
Physician	1	1	5
Pharmacist	1	1	0
Other	0	0	2

**#x002A;:** The hospital setting had 2 cohorts, the outpatient setting had 2 cohorts, and the long-term care setting had 3 cohorts.

## Discussion

This study is one of the first to focus on all three healthcare settings—hospitals, outpatient care, and nursing homes. It demonstrates that collaboration between antibiotic stewardship experts and non-academic settings are feasible and has significant potential to reach rural populations and vulnerable communities.^8^ It also further supports the concern that outpatient settings—which are numerous and highly varied—may be the most difficult to reach with antibiotic stewardship training and policy change.

Though the intervention had significant reach, it had several limitations to be addressed in future efforts. Participants attended webinars inconsistently, with uneven participation in discussions. Most participants were off camera, with some stating that their computers lacked cameras. Multi-tasking during sessions was also a barrier to discussion. Most input was limited to the chat function, even in small group sessions. Attempts at QI initiative presentations, including offering tote bags for volunteers, were largely unsuccessful though anecdotal feedback during sessions was positive. We conducted an individual consultation for a hospital participant; no others were requested. A decline in the number of LTCs in Year 3 may represent moderating demand for the program, though participant attendance was higher than in Year 2. The study itself was limited due to the nature of the funding and its prospective cohort design without a control group or clinical outcomes. Future efforts should strengthen implementation supports and assess how SVI impacts antibiotic stewardship.

Overall, efforts to improve antibiotic stewardship have often focused on only one or two settings—primarily hospitals, secondarily nursing homes, or on specific antibiotic stewardship issues and not on all core elements of antibiotic stewardship.^9,10^ NC CLASP used a small team of antibiotic stewardship experts to provide broad, statewide, systemic education and, while far from perfect in its scope and reach, represents an improvement over previous, less comprehensive efforts. Furthermore, such a program could be easily replicable in other states and has the potential to improve antibiotic stewardship across the spectrum of healthcare settings if augmented with other practice change initiatives.

## Supporting information

10.1017/ash.2025.10245.sm001 Kistler et al. supplementary material Kistler et al. supplementary material

## Supplementary material

For supplementary material accompanying this paper visit https://doi.org/10.1017/ash.2025.10245

## Research transparency and reproducibility

Access to data can be made available upon request.
