# Supplementary material for: NC CLASP: the structure and reach of a statewide antibiotic stewardship education program
Source: Antimicrob Steward Healthc Epidemiol. 2025 Dec 17;5(1):e345. doi: 10.1017/ash.2025.10245 (PMC12722555; doi:10.1017/ash.2025.10245)
Supplement: Kistler et al. supplementary material [file S2732494X25102453sup001.docx]

**Figure Aggregated Maps of Hospital, Outpatient Clinic, and Long-term Care Setting Sites with SVI Density Mapping**

**Figure 1a. Hospital Setting**

**
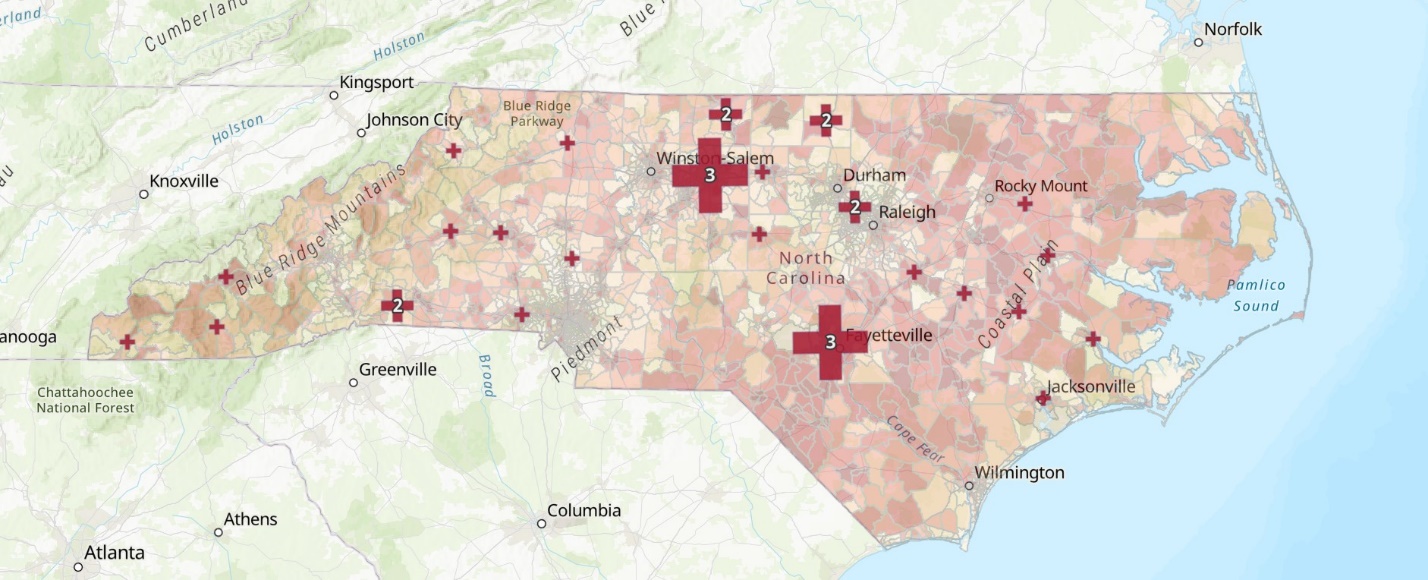
**

**Figure 1b. Outpatient Clinic Setting**

**
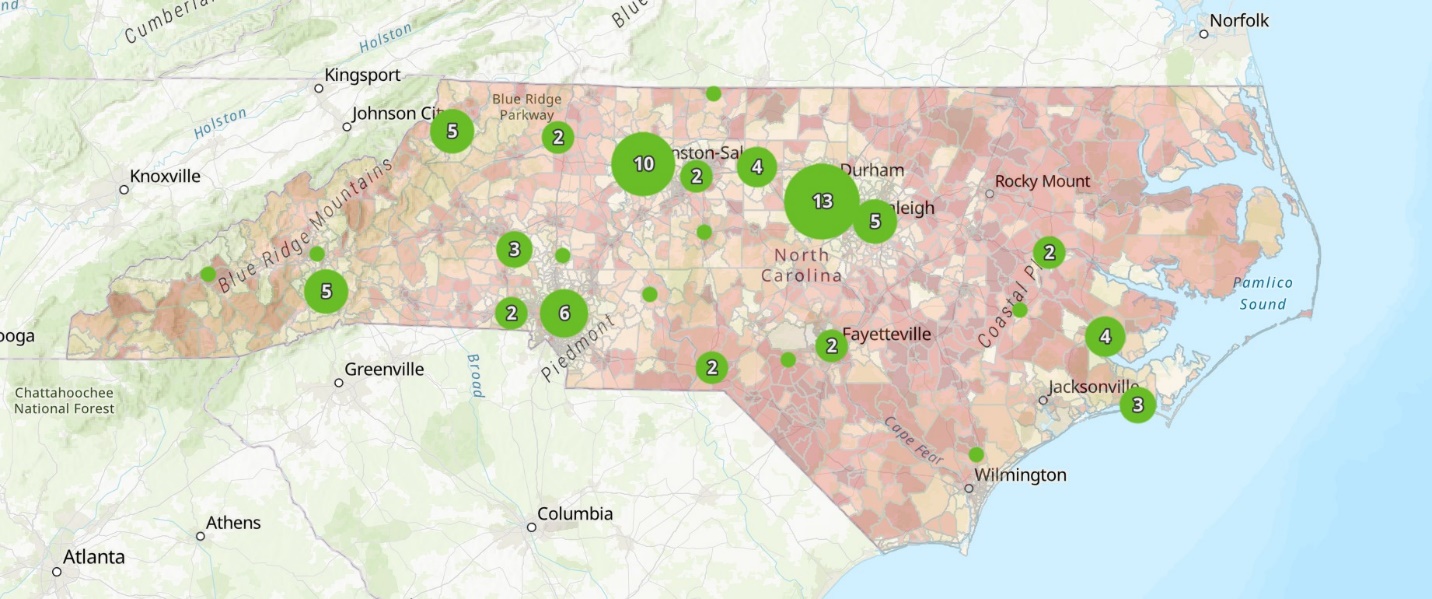
**

**Figure 1c. Long-term Care Setting**

**
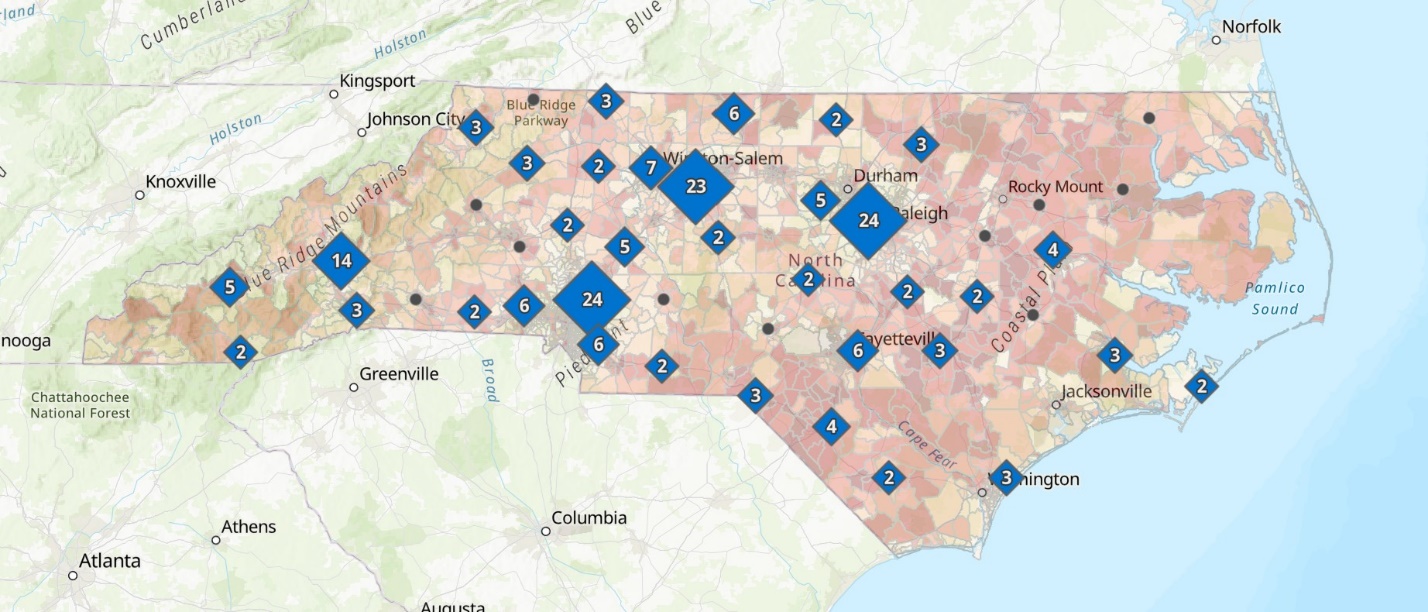
**

**SVI ranges from light pink to darker red, with the darker colors having higher SVI scores. Red crosses represent hospitals, green circles represent outpatient clinics, and blue diamonds represent nursing home communities.**

**0.000000-0.250000**

**0.250001-0.500000**

**0.500001-0.750000**

**0.750001-1.000000**

**Appendix**

| SESSION | SEQUENTIALLY | COHORT 1  ( ??-??) | COHORT 2  (??- 2/28/24) | COHORT 3 (3/6/24- 7/10/24) |
| --- | --- | --- | --- | --- |
| SESSION 1 | Intro to Core Elements | Intro to Core Elements + QI | Intro to Core + QI | Intro to Core + QI |
| SESSION 2 | #1 Leadership | Education + SMART AIMS | Accountability + Leading Change |  |
| SESSION 3 | #2 Accountability | Action + QI on Communication | Action + SMART AIMS |  |
| SESSION 4 | #3 Pharmacy Expertise | Tracking/Reporting + QI on T/R | Education + Crossing the know-do gap |  |
| SESSION 5 | #4 Action | Pharmacy Expertise + Outcome Assessment | Leadership + QI communication |  |
| SESSION 6 | #5/6 Tracking/Reporting | Leadership + PDSA | Tracking/Reporting + QI on T/R |  |
| SESSION 7 | #7 Education | Accountability + Leading for change | Pharmacy Expertise + Outcome Assessment. |  |
| SESSION 8 | Summary and Wrap-up | Summary and Wrap-up | Summary and Wrap-up | Summary and Wrap-up |
